# A Case Report of a Man with Burning Arm and Leg Weakness

**DOI:** 10.21980/J8V659

**Published:** 2022-10-15

**Authors:** Carolina Ornelas-Dorian, Paul Jhun

**Affiliations:** * University of California, San Francisco, Department of Emergency Medicine, San Francisco, CA

## Abstract

A 44-year-old male presented with sudden onset of severe left arm burning dysesthesia and bilateral leg numbness and weakness for several hours. He denied any recent illnesses or trauma and was previously healthy. His exam showed decreased strength to his left upper extremity, decreased light touch sensation to bilateral lower extremities, and urinary retention. Computed tomography (CT) and magnetic resonance imaging (MRI) of the cervical spine were performed, which demonstrated acute cervical myelopathy due to congenital cervical stenosis, a less common finding. Congenital cervical stenosis is the narrowing of the cervical spinal canal that is not due to structural, infectious, vascular, or malignant causes. This is an important diagnosis to consider in patients who present with neurologic symptoms without risk factors for common myelopathy causes (eg, degenerative changes). Early diagnosis and treatment are essential to prevent long term neurologic deficits.

**Topics:**

Neurosurgery, cervical myelopathy, acute neurologic deficits.

**Figure:**
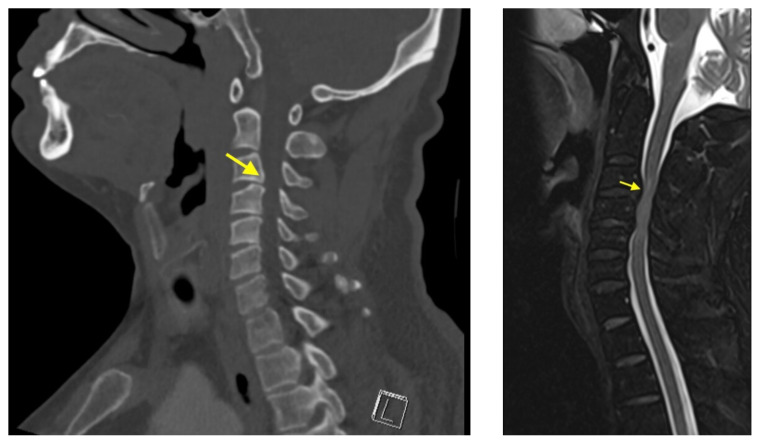


## Brief introduction

Cervical myelopathy is compression of the spinal cord in the setting of spinal stenosis (narrowing of the spinal canal). Cervical spine stenosis affects 5% of the general adult population (9% in individuals aged 70 or older).^1^ Diagnosis of cervical myelopathy is challenging, especially in the early stages when symptoms are vague and non-specific, such as neck pain and paresthesia.^2^ The differential diagnosis includes infectious (eg, spinal epidural abscess), structural (eg, degenerative, congenital), vascular (eg, vascular malformation), and malignant causes. Congenital cervical stenosis is a relatively uncommon etiology, with an unknown prevalence, that is limited to mainly case and autopsy reports.^3^,^4^ It is nevertheless critical to diagnose due to the risk of irreversible loss of neurologic function.^5^,^6^

## Presenting concerns and clinical findings

A 44-year-old male presented with sudden onset of severe left arm burning dysesthesia and bilateral leg numbness and weakness for several hours. He denied neck pain, preceding symptoms, recent illness, fever, or trauma. Physical exam of the left upper extremity revealed one out of five strength to his left bicep, tricep, and wrist extensors and flexors, as well as two out of five strength to his left finger flexors. The right upper extremity revealed four out of five strength diffusely. Lower extremity examination revealed four out of five strength to his bilateral hip and knee flexors and extensors, and five out of five strength to his bilateral dorsiflexors and plantar flexors. He reported decreased light touch sensation diffusely to bilateral lower extremities, and hyperesthesia to his left upper extremity. Though bilateral knee and ankle reflexes were difficult to elicit, he had symmetric 2+ bicep and brachioradialis reflexes bilaterally. Cranial nerve examination was normal. However, the exam demonstrated urinary retention, and he was unable to ambulate.

## Significant findings

A non-contrast computed tomography (CT) of the head and neck was performed, followed by an MRI of the cervical spine. The CT demonstrated congenital narrowing of the cervical spinal canal, with posterior disc osteophyte complex and disc bulge at C3–4 and C4–5 (arrow). The T2-weighted MRI additionally demonstrated obliteration of the anterior and posterior subarachnoid space at the level of C3–C5, with associated patchy central cord signal abnormality (arrow).

## Patient course

In the emergency department (ED), the patient underwent a broad workup for his symptoms. X-ray imaging of his left upper extremity was normal. His ECG and chest X-ray were normal. His blood work (venous blood gas, complete blood count, complete metabolic panel, creatinine kinase, coagulation factors, troponin, erythrocyte sedimentation rate, and C-reactive protein) was remarkable only for an elevated lactate (3.8 mmol/L) and mildly elevated creatine kinase (264 U/L). Urinalysis was normal. MRI of the brain demonstrated no acute infarct and no intracranial abnormality. Neurosurgery was consulted, and the patient underwent an emergent C3–C5 posterior decompression. The patient was hospitalized for 9 days and subsequently was transferred to an inpatient rehabilitation center, where he was admitted for 14 days. Once discharged, the patient participated in outpatient physical therapy for several weeks. Several months after his original diagnosis, the patient significantly improved, but he reported persistently mild decreased sensation to his left upper extremity and four out of five strength in his left interosseous muscles. The other extremities recovered full strength. His urinary retention resolved, and he was able to ambulate independently.

## Discussion

Emergency medicine clinicians should maintain a wide differential diagnosis and perform a broad workup to determine the cause for patients presenting with acute, focal neurologic abnormalities. Based on the history and exam, advanced imaging, such as CT and MRI imaging of the brain and spine, is an important diagnostic tool in evaluating etiologies. Acute cervical myelopathy is commonly due to acquired causes, such as degenerative changes, malignancy, or trauma. However, in the absence of significant risk factors, as in this case, clinicians must consider congenital causes, such as congenital cervical stenosis. Congenital cervical stenosis is the constriction of the cervical spinal canal that is not due to “degenerative, pathologic, dysplastic, or deforming changes.”^1^ Cervical stenosis is classified through the Muhle classification system according to the degree of “obliteration of the anterior or posterior subarachnoid space, and the presence of cervical cord compression or displacement.”^5^,^7^

Acute cervical myelopathy is a neurosurgical emergency with the potential for permanent neurologic damage. Management is primarily surgical, with a posterior cervical laminectomy. Neurosurgeons remove the spinal laminae to ease acute compression on the spinal cord.^6^

MRI is the gold standard for diagnosing acute cervical myelopathy, with a sensitivity of 79–95% and specificity of 82–88%.^3^ On the other hand, CT has a sensitivity of 82% and specificity of 68%.^8^ It is excellent at detecting fractures (sensitivity 97–100%), but is inferior to MRI at detecting ligamentous and spinal cord lesions.^9^ In one study, 92% of cervical myelopathy cases (diagnosed with MRI) were detected on CT. However, 51% of these cases had cord atrophy, which is more accurately detected through MRI.^10^

One limitation of this case report centers on the accessibility of MRI. For ED clinicians practicing where MRI is not widely available, CT imaging can be obtained. However, when suspecting acute cervical myelopathy, clinicians must be aware of the diagnostic limitations of CT and transfer patients in a timely manner.

In summary, congenital canal stenosis can lead to acute cervical myelopathy in patients who are otherwise healthy. Patients can present with a range of neurologic signs and symptoms, including weakness, numbness, hyperesthesia, pain, and urinary retention. Acute cervical myelopathy is a neurosurgical emergency that requires MRI for definitive diagnosis and surgical management.

## Supplementary Information








