# Supplementary figures and images for: A Case Report of a Man with Burning Arm and Leg Weakness
Source: J Educ Teach Emerg Med. 2022 Oct 15;7(4):V4–6. doi: 10.21980/J8V659 (PMC10332663; doi:10.21980/J8V659)

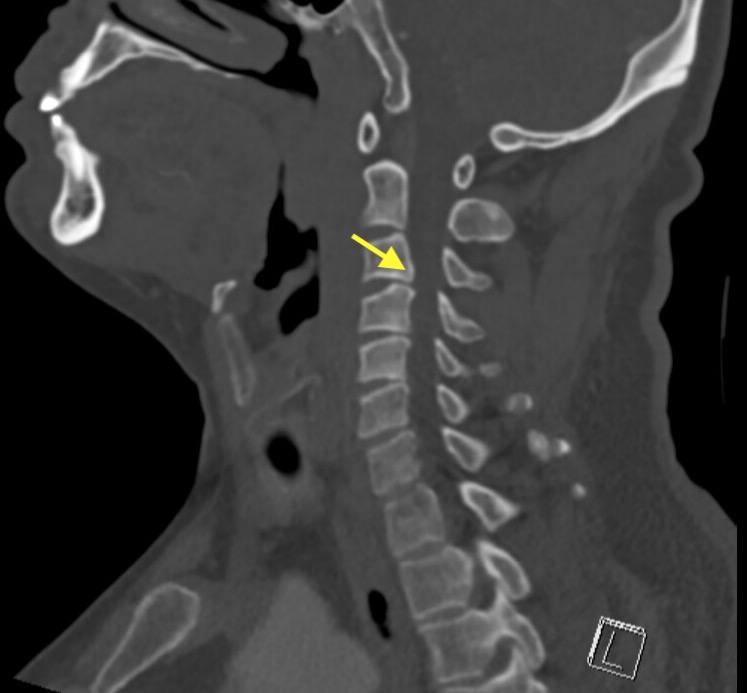

Supplement: Supplementary file 1 [file JETem-7-4-V4-supp1.jpeg]

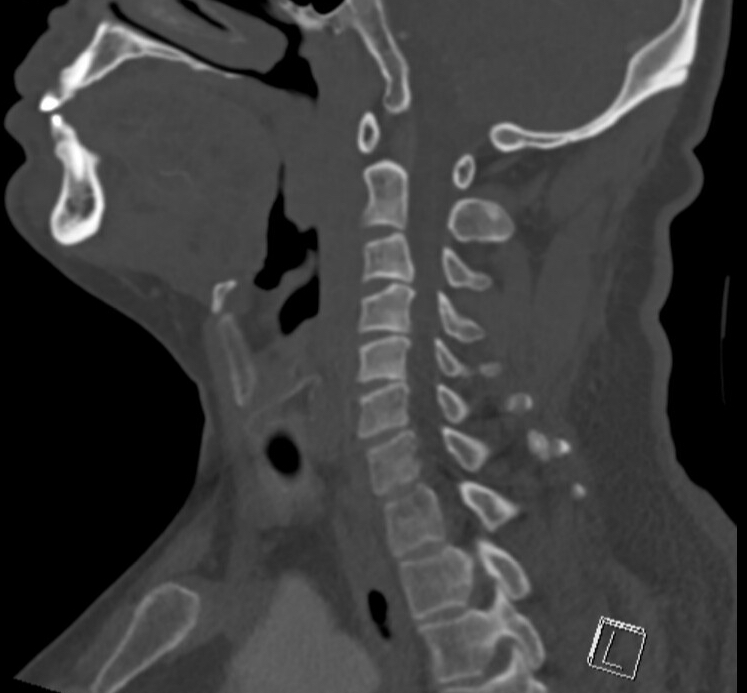

Supplement: Supplementary file 2 [file JETem-7-4-V4-supp2.jpeg]

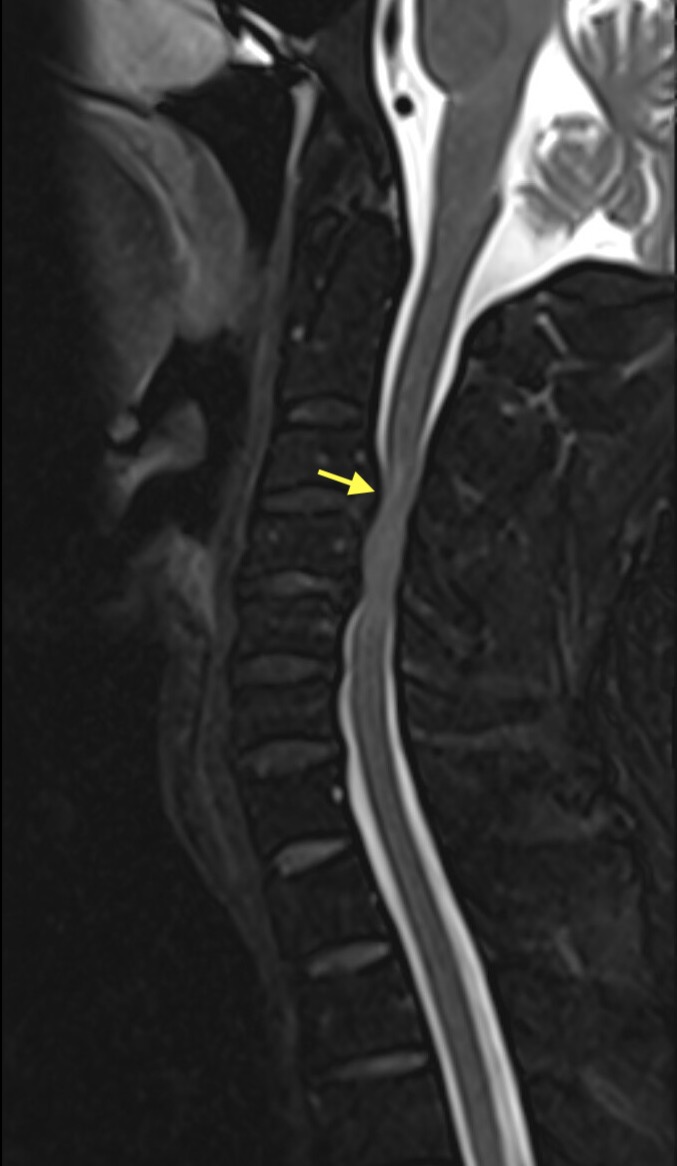

Supplement: Supplementary file 3 [file JETem-7-4-V4-supp3.jpeg]

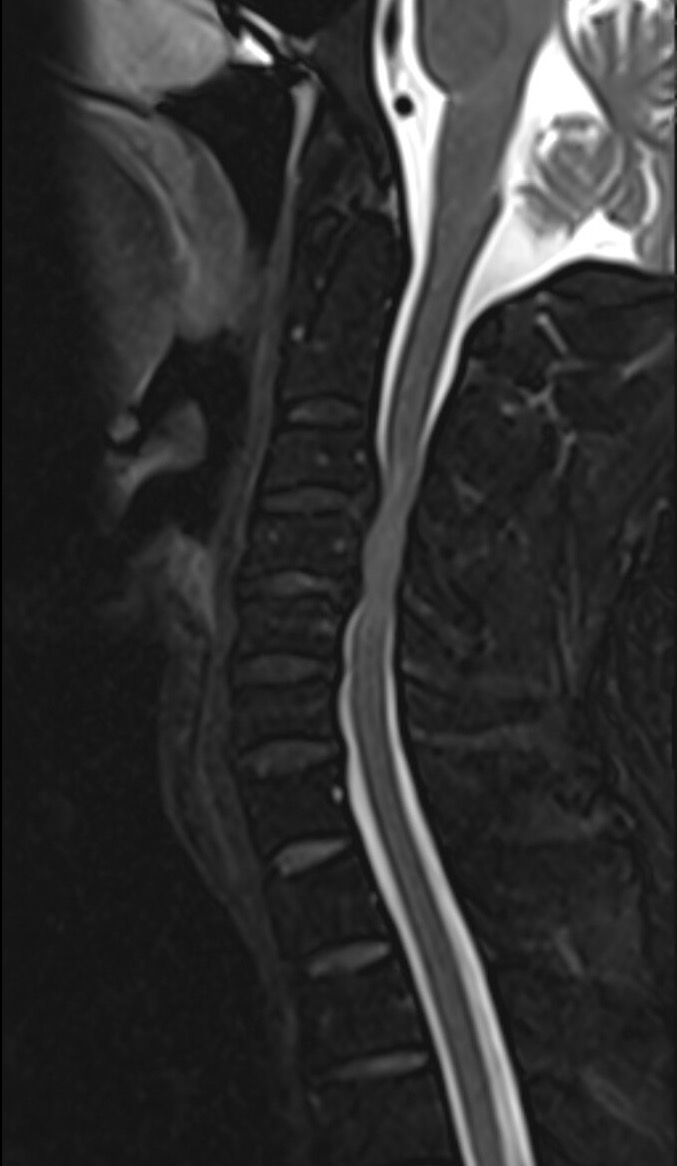

Supplement: Supplementary file 4 [file JETem-7-4-V4-supp4.jpeg]
